# Prediction of Ground Wave Propagation Delay for MF R-Mode

**DOI:** 10.3390/s24010282

**Published:** 2024-01-03

**Authors:** Niklas Hehenkamp, Filippo Giacomo Rizzi, Lars Grundhöfer, Stefan Gewies

**Affiliations:** Deutsches Zentrum für Luft- und Raumfahrt (DLR), Institute of Communication and Navigation, 17235 Neustrelitz, Germany; filippo.rizzi@dlr.de (F.G.R.); lars.grundhoefer@dlr.de (L.G.); stefan.gewies@dlr.de (S.G.)

**Keywords:** R(anging) -mode, ground wave propagation, radionavigation

## Abstract

Time delays caused by ground wave propagation are the primary source of systematic error limiting the performance of the medium-frequency R-Mode radionavigation system. To achieve the desired ranging accuracy and compensate these delays, we have conceived a comprehensive correction scheme based on the prediction and application of the Atmospheric and Ground wave Delay Factor (AGDF). The AGDF was computed and mapped in 2D for a number of MF R-Mode transmitters in the Baltic Sea that were embedded into the receiver and evaluated during a large-scale measurement campaign. Our results show that the proposed AGDF approach is valid for the MF R-Mode system and provides accurate corrections of ground wave propagation delays within the performance requirements.

## 1. Introduction

Ranging Mode, known as R-Mode, is a maritime terrestrial radio navigation system currently under development [1,2,3,4,5,6,7]. It is intended to reduce the dependence of today’s maritime transport on global navigation satellite systems (GNSS). Furthermore, as an alternative navigation system, it is intended to be available in case GNSS fails, as well as to increase the overall availability of positioning, navigation, and timing (PNT) information for maritime applications with higher demands on PNT continuity, availability, and integrity.

R-Mode is designed as a cost efficient extension of current maritime radio communication infrastructure. Land-based transmitters of that infrastructure are extended by highly accurate timing sources that enable the broadcast of R-Mode network-wide synchronized timing signals, thereby enabling range estimation between the transmitter and the mobile receivers. The reception of three R-Mode transmitting stations enables positioning and timing.

Currently, the implementation of R-Mode is under investigation on medium-frequency (MF) radio beacons [1,2,4,6], that broadcast differential GNSS corrections in coastal regions within distances of about 250 km from transmitters ashore as well as on base stations of the very high frequency data exchange system (VDES) [7,8,9,10], which is used for different maritime services such as dynamic and static ship information or ship route exchange. VDES and MF R-Mode work in different frequency bands, which causes a difference in the signal propagation. For VDES, the signals have to fulfil direct line-of-sight conditions in order for it to be used for positioning and timing. For MF, the main propagation path is a ground wave. This paper focuses on the MF component of R-Mode.

Initial theoretical analysis and measurements in different testbeds have demonstrated the feasibility of MF R-Mode as a backup for GNSS in the maritime domain [1,2,4]. A positioning performance of 10 m could be achieved during daytime in areas with a nearly constant propagation path related to the composition of land and sea [4]. During the night, the sky wave reduces performance for distant stations, which currently reduces the usability of signals to distances of about 70 km from MF R-Mode transmitters [1]. Countermeasures that suppress the sky wave propagation path at night are under investigation.

To support ranging with the radio beacon transmission, two continuous wave (CW) aiding carriers are added next to the edges of the 500 Hz to 1 kHz wide channel of each radio beacon [11] located in the maritime frequency band from 283.5 kHz to 325 kHz [12]. Measuring the phase of both CW aiding carriers allows the ambiguities to be solved with the help of the beat signal (both carriers of one station) and the range to be estimated within the last incomplete wave length (about 0 to 1000 m). An essential basic requirement when transferring the phase information to the distance is exact knowledge of the signal propagation.

Medium-frequency R-Mode signals experience significant ground wave propagation delays caused by the finite ground conductivity and relative permittivity of the surface. As in the low-frequency-based LORAN-C and eLoran radio navigation systems, these delays are one of the most influential error sources in the system, and can cause a large systematic decrease in the horizontal positional accuracy of MF R-Mode.

To compensate for the effects of ground wave propagation and atmospheric delays, we developed a method to predict and correct the propagation delay of MF R-Mode signals, called the Atmospheric and Ground Wave Delay Factor (AGDF) [13,14]. This paper is the first detailed description and performance analysis of the approach.

The paper is organized as follows: Section 2 describes the mathematical framework used for the calculation of ground wave propagation delays, while Section 3 introduces the AGDF and explains how it is computed. In Section 4, we present the results of a large-scale measurement campaign that was conducted in the Baltic Sea MF R-Mode testbed and evaluate the performance of the AGDF prediction with regard to the improvement of R-Mode ranging accuracy. Finally, in Section 5 we discuss the results of the performance evaluation and provide an outlook of future activities that are planned or have been proposed for the improvement of the solution.

## 2. Ground Wave Propagation

In this section, we introduce the mathematical foundations upon which the calculation and prediction of ground wave propagation delays are based in the proposed AGDF approach for the MF R-Mode system.

The effect of ground wave propagation in the long- and medium-frequency band has been discussed extensively in the literature. Wait [15] provided a detailed overview of the evolution of theories related to electromagnetic ground wave propagation. The attenuation of a wave travelling along the interface of the earth’s surface and the atmosphere is caused by the finite complex surface impedance, consisting of the dielectric permittivity ϵ and the conductivity σ of the ground. The term “attenuation” is a complex valued factor, involving the introduction of an amplitude damping and a phase delay in addition to the free space propagation loss of a wave. To obtain the AGDF for the MF R-Mode system, we want to derive a method to calculate the phase of the complex attenuation function.

A comprehensive description and discussion of the theoretical foundations and calculation methods of electromagnetic ground wave propagation can be found in [16]. In brief, the electric field of a ground wave can be expressed by multiplying the vertical electric field in free space $E_{0}$ in (1) for a time-variant vertical electric dipole source of moment Ids

(1) $$E_{0}=-j\mu_{0}\omega \mathrm{Ids}{\left(2\pi r\right)}^{-1}\exp\left(-jkr\right),$$

where ω is the angular frequency, *k* is the wave number, $\mu_{0}$ is the vacuum magnetic permeability, and r is the distance from the source, with an attenuation function W in

(2) $$E=E_{0}W.$$

For relatively short distances with respect to the wavelength, the approach of modelling the earth as a plane surface according to [17,18] can be applied to calculate the electric field of a ground wave within a certain margin of accuracy. The resulting attenuation function W(p) can be calculated through

(3) $$W\left(p\right)=1-\sqrt{\pi p}e^{-p}\mathrm{erfc}\left(j\sqrt{p}\right),$$

where

(4) $$\mathrm{erfc}\left(j\sqrt{p}\right)=\frac{2}{\sqrt{\pi }}\int_{j\sqrt{p}}^{\infty }\exp\left(-z^{2}\right)dz$$

and the numerical distance *p* is

(5) $$p=\frac{-jkr}{2N^{2}}$$

with the complex refractive index of the half-space *N* being

(6) $$N^{2}=\frac{\sigma +j\epsilon \omega }{j\epsilon_{0}\omega }.$$

The above solution is valid under the assumption that

(7) $${\left|N\right|}^{2}>>1.$$

For larger distances, the earth’s curvature is taken into account by employing the residue series solution of the problem, which converges poorly at shorter distances [15].

Following the detailed derivation presented in [16], the attenuation function of a vertically polarized ground wave at the great circle distance *d* from a vertical electric dipole source of moment Ids propagating along a curved earth can be expressed through the following series:

(8) $$W\left(x\right)={\left(\pi xj^{-1}\right)}^{\frac{1}{2}}\sum_{s=1,2,3,...}^{\infty }\frac{G_{s}\left(y_{a}\right)G_{s}\left(y_{b}\right)\exp\left(-jxt_{s}\right)}{t_{s}-q^{2}},$$

where

(9) $$q=-j(\frac{ka}{2}{)}^{\frac{1}{3}}\Delta$$

and

(10) $$x=(\frac{ka}{2}{)}^{\frac{1}{3}}\left(\frac{d}{a}\right),$$

with Δ being the complex surface impedance of the earth Z normalized by 120π, $G_{s}$ the Height-Gain Function including refraction in a nonlinear atmosphere, *y* the transmitter/receiver height (assumed to be 0), *x* the numerical distance, *a* the effective earth radius, and *k* the wave number. A detailed description of the effective earth-radius concept as well as the nonlinear atmosphere representation in the Height-Gain functions can be found in [16]; $t_{s}$ are the roots of the mode in Equation (11) involving the Airy integral function w(t):

(11) $$w^{\prime }\left(t\right)-qw\left(t\right)=0.$$

As a response to the poor convergence of the residue series at short distances and the inaccuracy of the flat-earth solution with respect to the effect of the earth’s curvature at low frequencies, a modified rapidly converging series solution proposed by [19] can be used for short distance calculations.

A well-established approach to calculating the attenuation of a ground wave is to use a hybrid solution that employs the residue series for larger distances from the transmitter and the flat-earth solution with the power series expansion presented by [20] for shorter distances, with the attenuation function

(12) $$W\left(x\right)=\sum_{m=0}^{10}A_{m}{\left[\exp\left(j\pi /4\right)qx^{1/2}\right]}^{m}.$$

where

(13) $$\begin{array}{c} A_{0}=1,\\ A_{1}=-j\sqrt{\pi },\\ A_{2}=-2,\\ A_{3}=j\sqrt{\pi }\left(1+1/\left(4q^{3}\right)\right),\\ A_{4}=4/3\left(1+1/\left(2q^{3}\right)\right),\\ A_{5}=-j\sqrt{\pi }/4\left(1+3/\left(4q^{3}\right)\right),\\ A_{6}=-8/15\left(1+1/q^{3}+7/\left(32q^{6}\right)\right),\\ A_{7}=j\pi /6\left(1+5/\left(4q^{3}\right)+1/\left(2q^{6}\right)\right),\\ A_{8}=j\sqrt{\pi }/6\left(1+5/\left(4q^{3}\right)+1/\left(2q^{6}\right)\right),\\ A_{9}=-j\sqrt{\pi }/24\left(1+7/\left(4q^{3}\right)+5/\left(4q^{6}\right)+21/\left(64q^{9}\right)\right),\\ A_{10}=-1\left(32/945+64/\left(945q^{3}\right)+11/\left(189q^{6}\right)+7/\left(270q^{9}\right)\right) \end{array}$$

Finally, the R-Mode AGDF is based on the phase delay $\phi_{\mathrm{GW}}$ of the ground wave over a homogeneous propagation path with respect to free space propagation in vacuum, and is given as

(14) $$\phi_{\mathrm{GW}}=-\arg\left(W\left(x\right)\right).$$

Several equivalent solutions can be found in the literature. The equations that are presented in [21] can be implemented quickly and used for comparison, showing that the different approaches are in good agreement. The LFMF software package recommended by ITU-R P.368-10 [22] considers an atmospheric refraction index with exponential decay, implements the aforementioned equations, and is written in C++, available under a permissive license. The results computed using LFMF account for the effect of finite ground conductivity and atmospheric propagation at an accuracy beyond linear or polynomial approximations. For computation of the AGDF, we modified LFMF to calculate the complex attenuation, i.e., the amplitude and phase delay, and wrapped it in the Python programming language.

**Figure 1:**
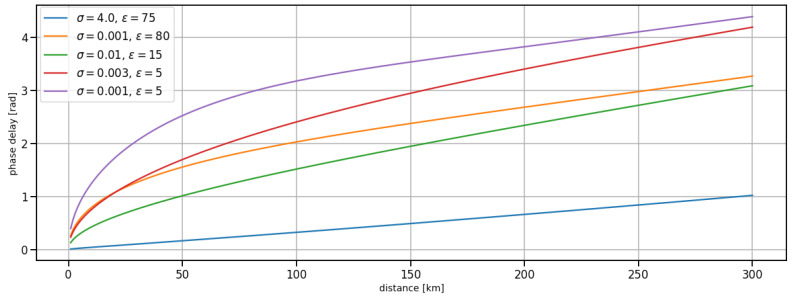
Argument of the attenuation function *W* (ground wave phase delay) at f = 300 kHz for different values of ground conductivity σ and permittivity ϵ; blue: sea water, green: rich agricultural land, orange: fresh water (low salinity), red: moderately good soil, purple: dry ground.

Figure 1 depicts the ground wave phase delay over distance for selected ground types at a frequency of 300 kHz as calculated using the modified version of LFMF created within the scope of this work. The plot represents the ground conductivity and permittivity of different ground types for the typical range of an MF R-Mode transmitter. While the phase delay introduced by ground wave propagation across seawater increases almost linearly over distance with a comparably small slope, propagation across land introduces large phase delays that vary significantly depending on the ground conductivity of the surface. Over the nominal range of an MF R-Mode signal, the phase delay caused by ground wave propagation is on the order of up to half a wavelength.

For a non-homogeneous path comprising multiple sections with different surface impedance, the attenuation function has to account for the discontinuity of electrical properties. Thus, the strong change of the wave tilt causes changes in amplitude and phase along the propagation path.

The attenuation function $W_{P}$ of a wave travelling along a perturbed path of variable surface impedance can be calculated with the integral equation method [23,24] based on the Volterra-type integral equation of the second kind:

(15) $$W_{P}\left(d\right)=W_{0}\left(d\right)-{\frac{jd}{\lambda_{0}}}^{1/2}\int_{0}^{d}\left(\Delta_{s}\left(d\right)-\Delta_{e}\right)W_{P}\left(y\right)W_{0}\left(d-y\right)\sec\left(\alpha \right)\frac{dy}{\left[y(d-y)\right]}$$

where *d* is the distance from the transmitter, α is the slope angle of the terrain at a given point, $W_{0}\left(d\right)$ is the attenuation function for a homogeneous path with the reference normalized surface impedance $\Delta_{0}$, and $\Delta_{s}\left(d\right)$ is the varying normalized surface impedance of the perturbed propagation path as a function of distance and the equivalent normalized surface impedance

(16) $$\Delta_{e}=\Delta_{0}\cos\left(\alpha \right)-\sin\left(\alpha \right).$$

The numerical solution of the integral equation proposed by Monteath [25] is implemented in the Python programming language within the scope of this work. The attenuation function $W_{0}\left(d\right)$ for a homogeneous path with the reference normalized surface impedance $\Delta_{0}$ is computed using the modified version of the LFMF software, using a value for $\Delta_{0}$ that represents propagation across an all-seawater path with a ground conductivity σ = 4 mS/m and a relative dielectric permittivity $\epsilon_{r}$ = 80.

**Figure 2:**
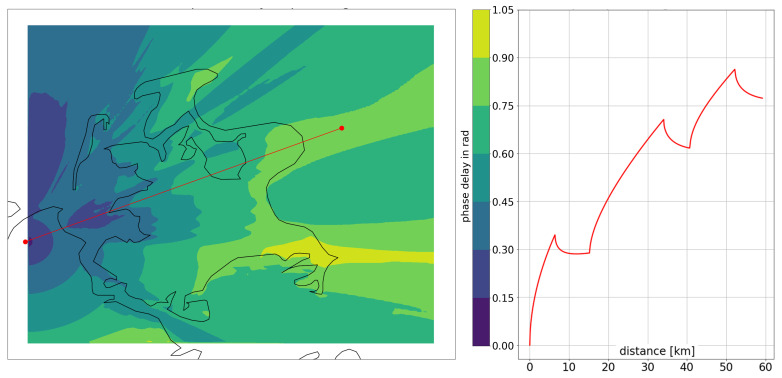
(**Left**) Two-dimensional map of the phase delay of the ground wave with respect to free space in the area of the German island of Rügen. The red line illustrates an example path between the R-Mode transmitter (left red dot) and a hypothetical vessel position east of the island. (**Right**) Ground wave phase delay for the same propagation path over distance.

Figure 2 depicts the mixed-path ground wave phase delay of the MF R-Mode transmitter Groß Mohrdorf for a typical propagation path across the island of Rügen and the Baltic Sea. The effect of phase recovery at the boundary between land and sea is visible in the right half of the picture. The varying proportion of land along the overall composition of the propagation path results in variant shading of the ground wave propagation delay along the coastline. The phase of the signal varies on the order of up to an eighth of a wavelength around the island along an arc at a constant distance to the transmitter.

The largest source of error in the prediction of ground wave propagation delays is the inaccuracy of the underlying ITU-R P.832-3 World Atlas of Ground Conductivities [26]. There are simply no data available for many regions, and where data exist they are often based on sparse measurements, which in the case of Germany were obtained decades ago.

## 3. MF R-Mode Atmospheric and Ground Wave Delay Factor (AGDF)

To compensate for the effect of ground wave propagation delays, a correction scheme that involves the calculation and 2D prediction of the ground wave attenuation function has been conceived. Based on Equation (2), the measured electric field of the R-Mode signal can be expressed as the product of the vertical electric field in free space $E_{0}$ (defined in (1)) and the ground wave attenuation function *W* for the propagation path. To obtain ranges from phase estimates with respect to the speed of light in a vacuum $c_{0}$, the argument of the attenuation function has to be subtracted from the measured field. The Atmospheric and Ground Wave Delay Factor (AGDF) is defined as the ground wave phase delay $\phi_{GW}$ or the negative argument of *W* (see Equation (16)), and has to be added to the estimated phase of the measured signal, as in

(17) $$arg\left(E_{0}\right)=arg\left(E\right)+\phi_{GW},$$

to obtain the propagation delay of the free space wave.

The AGDF accounts for the effect of finite ground conductivity and the atmospheric delay between transmitter and receiver. In practice, the aforementioned effects can neither be modelled nor predicted completely. The temporal variation of the AGDF caused by changes in temperature, soil moisture, ice coverage, and other factors is not predictable on the basis of static ground conductivity maps. The deviation of the true ground electrical characteristics and the static map leads to an additional error. Additionally, there is an unknown delay that occurs in the transmitter and the receiver. Equation (18) displays the problem by separating the AGDF into a predicted part $AGDF_{p}$, a temporal part $AGDF_{t}$, and an error caused by map inaccuracies $AGDF_{e}$, then adding the transmitter $\phi_{TX}$ and receiver delay $\phi_{RX}$ as well as a residual phase error $\phi_{r}$ that accounts for noise and higher-order effects we cannot describe with the approach [27]:

(18) $$arg\left(E_{0}\right)=arg\left(E\right)+\left(AGDF_{p}+AGDF_{t}+AGDF_{e}\right)-\left(\phi_{TX}+\phi_{RX}+\phi_{r}\right).$$

In summary, adding the predicted $AGDF_{p}$ to the estimated phase of the received signal yields the free space propagation delay plus the transmitter delay, receiver delay, temporal AGDF variation, map inaccuracy, and a residual error. The $AGDF_{p}$ is predicted based on the equations presented in Section 2 and a static database of ground conductivities. This accounts for ground wave phase delay and atmospheric delay. The ratio of the predictable part $AGDF_{p}$ and unpredictable part of the AGDF, which contains $AGDF_{t}$, $AGDF_{e}$, and $\phi_{r}$, cannot be quantified easily. In general, the quality of the prediction, and as such the fraction of the AGDF that is predictable, depends on the quality of knowledge about the earth‘s surface. If the ground conductivity, ground electrical permittivity, and terrain features are well known in the sense of high geometric resolution and a full description of the spatial and temporal dynamics, the prediction is theoretically bound by the uncertainty of the integral equation approach, which is based on the assumption that ground topography is slowly varying [27].

### 3.1. Calculation of the $AGDF_{p}$

For the determination of the $AGDF_{p}$ for MF R-Mode, LFMF software was modified to compute the phase and amplitude of the complex attenuation function $W_{0}$ with a reference normalized surface impedance $\Delta_{0}$ representing seawater. For a non-homogeneous mixed path, the normalized surface impedance of the path $\Delta_{s}$ is calculated using the ground conductivity σ obtained from ITU-R P.832-3 in conjunction with reasonable values of the relative permittivity $\epsilon_{r}$.

Because the data in ITU-R P.832-3 is not provided in a machine-readable format, it was converted into a shapefile that contains polygons of equal conductivity and a high-resolution map of coastlines. For the calculation of $AGDF_{p}$, the propagation path segments were determined by an intersecting propagation path with the polygons and calculating the intersection point with each polygon using the Python packages Geopandas and Shapely. The slope of the terrain was determined by extracting elevation values from the EUDEM digital elevation model of Europe [28].

Using the Monteath method with an integration interval of 50 m, the $AGDF_{p}$ was calculated for the segmented path. The process of determining the intersection points of the polygons and the propagation path is both computationally demanding and time consuming. Therefore, the selected area of interest on the ground conductivity map was divided into a grid of evenly spaced points, for which the $AGDF_{p}$ computation was carried out once. The grid spacing may be larger in open sea areas with smaller $AGDF_{p}$ gradients, while the accuracy requirements for R-Mode in coastal areas and the strong influence of the land–sea boundary on the ground wave phase delay demands a smaller grid spacing near the coastline. The $AGDF_{p}$ for an arbitrary point within the area was then obtained by performing grid interpolation on a subset of points using Python Scipy (based on a Clough–Tocher scheme [29]).

### 3.2. Application of the $AGDF_{p}$

**Figure 3:**
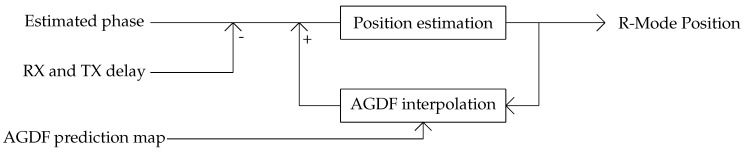
Simple feedback loop to apply AGDF corrections in the MF R-Mode receiver. Input: estimated phase of R-Mode signals, transmitter delay, and AGDF prediction map. Output: R-Mode position. The transmitter bias/delay is calculated in the initialization step, and could be provided by a supplementary R-Mode correction service that incorporates measurements from reference receivers.

The $AGDF_{p}$ is embedded into the receiver through the simple correction scheme shown in Figure 3, which is is added to the raw phase estimate after the transmitter delay is removed. A feedback loop is used to approximate the position, converging to a more accurate AGDF in each iteration.

AGDF maps of several transmitters were computed for the MF R-Mode testbed in the southern Baltic Sea. The figures in Appendix A depict the AGDF in radians for each of these transmitters. Table 1 contains the transmitter name, frequency, and location.

### 3.3. Comparison to LORAN ASF

In the LORAN-C and eLoran systems, the effects of ground wave propagation and atmospheric delay are compensated using a correction term, the propagation delay $T_{p}=PF+SF+ASF$, where PF is the primary phase factor, SF is the secondary phase factor, and ASF is the additional secondary phase factor. A detailed description of the propagation delay prediction and compensation techniques for Loran can be found in [30,31,32,33].

The PF accounts for atmospheric delay [34], while SF accounts for the propagation delay of the signal occurring over a homogeneous seawater path (ϵ = 70, σ = 5 mS/m). In general, the SF can be calculated with the methods described in Section 2, while for LORAN-C it is approximated with a polynomial. In practice, the effects of pure seawater propagation and mixed land–sea path propagation are hard to distinguish. The SF and ASF can be predicted or measured together and separated by subtracting the ground wave phase delay for seawater parameters [33]. The ASF accounts for all additional delays related to mixed-path ground wave propagation with respect to propagation across seawater. It can either be predicted with a certain accuracy, or measured systematically in survey campaigns. BALOR ASF prediction software can be used to predict the PF, SF, and ASF for an area based on a database of ground conductivity [31]. If an ASF is not available for a certain area, the correction of PF and SF still yields more accurate results than an uncorrected range estimate based on the free space propagation assumption.

Because the electrical parameters of the ground depend on moisture and temperature, there is a temporal variation of the ASF, as its magnitude depends on the specific area of interest [33]. The quality of ASF predictions always depends on the accuracy of the underlying ground conductivity and permittivity database. Therefore, the ASF can be expressed as a sum of the components

(19) $$ASF=ASF_{p}+\Delta ASF_{p}+\Delta ASF_{t},$$

where $ASF_{p}$ is the predicted ASF, $\Delta ASF_{p}$ is the error of the prediction introduced by database inaccuracies, and $\Delta ASF_{t}$ is the temporal variation. In eLoran, $\Delta ASF_{t}$ is measured and computed at reference sites. The obtained value is distributed through a differential eLoran service.

The AGDF of MF R-Mode signals is computed with a similar methodology to that of the ground wave propagation delay for Loran signals. Due to the differences in system design, MF R-Mode uses a correction scheme that is intended to compensate the delays for each CW tone of the signal on the phase level, rather than explicitly in the time domain. Table 2 lists the major differences between R-Mode and Loran.

The exact prediction and compensation technique used in Loran is not directly applicable to MF R-Mode. The AGDF is frequency-dependent and has to be determined for each transmitter individually, which would require a different polynomial to express the SF for each CW tone, while the seawater reference of the Loran SF is not applicable to inland waterways or bodies of brackish water such as the Baltic Sea. Because the ground wave propagation delay and atmospheric delay can be calculated all at once, the separation of these factors is not considered useful at the moment.

## 4. AGDF Performance Evaluation

The following section presents the results that were obtained during a four day measurement campaign conducted in the MF R-Mode testbed in the Baltic Sea. The data are used to highlight the effect of ground wave propagation delays on the ranging and positioning performance of the system and to evaluate the performance of the proposed correction scheme as well as the quality of the predictions made by our software.

### 4.1. Dynamic Measurements in the Baltic Sea R-Mode Testbed

The MF R-Mode receiver hardware [4,35] was installed on the ship Fyrbygarren provided by the Swedish Maritime Administration. During the voyage of the vessel between 28th and 31st August 2020, four MF R-Mode transmitters were in reception range (see Table 1), allowing for range and position estimation.

To assess the performance of ground wave phase delay corrections using the predicted R-Mode AGDF, we selected three different samples of the journey during which the impact of ground wave propagation was expected to cause significant disturbance of range estimation.

**Figure 4:**
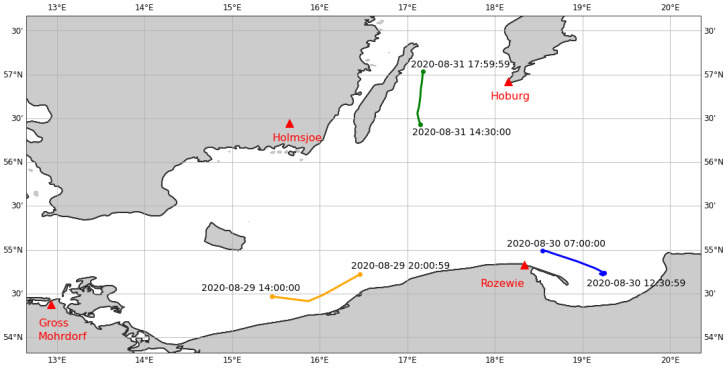
Ground track of the ship “Fyrbygarren” in the Baltic Sea during the MF R-Mode measurement campaign on August 29th (**orange**), 30th (**blue**), and 31st (**green**), 2020. The MF R-Mode transmitters that were used are marked as red triangles.

Figure 4 depicts the PPP reference track of the ship for each section.

In the following sections, the results of the measurements conducted on each day are presented according to the following structure: first, we compare the density histogram of the range error and the absolute range error for the uncorrected dataset relative to the speed of light in a vacuum $c_{0}$ without AGDF correction to the corrected dataset with AGDF correction. We evaluate its histogram and provide a table listing the mean, standard deviation, and 95th percentile of the absolute range error, used as performance indicators. Presenting both the range error and the absolute range error yields the possibility of better understanding the effect of AGDF correction. We highlight the signal with the most significant impact of ground wave phase delay on the ranging error by showing the respective AGDF map and the individual range error over time. Lastly, the effect of the range error and AGDF correction on the positional accuracy is shown over time together with a histogram depicting the density histogram of the ranging error for the corrected and uncorrected cases.

#### 4.1.1. First Dataset: Polish Coast—29 August 2020

**Figure 5:**
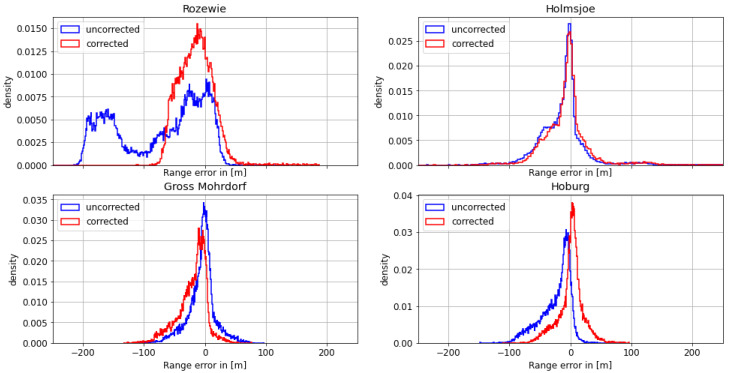
Histogram of the range error for the MF R-Mode transmitters in the testbed on **29 August 2020** with and without AGDF. **Blue**: no AGDF applied, ground wave phase delay not corrected. **Red**: AGDF applied, ground wave phase delay corrected.

**Figure 6:**
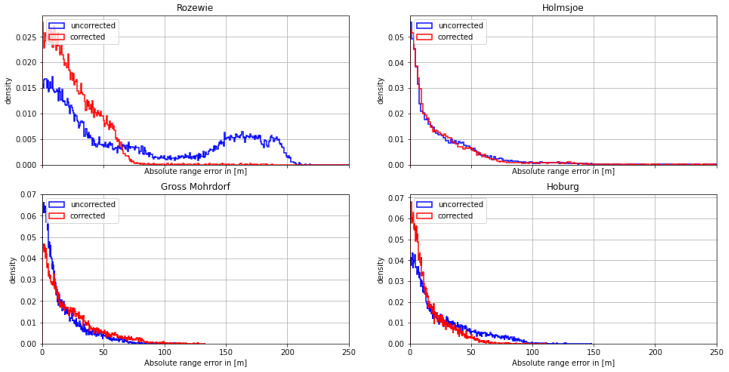
Histogram of the absolute range error for the MF R-Mode transmitters in the testbed on **29 August 2020** with and without AGDF. **Blue**: no AGDF applied, ground wave phase delay not corrected. **Red**: AGDF applied, ground wave phase delay corrected.

On August 29th, the Fyrbygarren was moving southwards from Bornholm into Polish waters, following a track eastward along the Polish coast. We obtained relatively satisfying results with respect to the overall ranging and positional accuracy. However, the range error of the signal from the transmitter in Rozewie was disturbed, presumably by ground wave phase delay. Figure 5 depicts the histogram of the range error for each of the transmitters. The range error density of the Rozewie transmitter is biased, with one maximum around a range error of −20 m and another maximum at around −160 m. The range error density of the corrected signal is still biased, though it has a smaller standard deviation and significantly smaller range error in the 95th percentile (see Table 3). It can be seen in Table 3 and Figure 6 that the absolute error decreases for all transmitters except Gross Mohrdorf. There, the AGDF correction does not lead to an improvement. In the case of the other transmitters, the signal from Hoburg is affected by the AGDF correction, while the Holmsjoe transmitter did not experience significant propagation path changes. In the case of Hoburg, the improvement resulting from the application of AGDF correction can be attributed to accurate compensation of the delay caused by propagation across seawater. The shift of the uncorrected density plots to the left in the cases of Hoburg and Holmsjoe is caused by the negative range error due to the decreased velocity of the signal across seawater compared to free space vacuum propagation together with the movement of the vessel towards the transmitter. In the case of Gross Mohrdorf, the AGDF caused an overcompensation that can be attributed to the inaccurate modelling of seawater propagation delay, possibly because of increased seawater salinity in the western Baltic Sea in comparison to the ITU-R P.832 reference.

In addition to the improvement due to the AGDF in the case of the Rozewie transmitter, Table 3 shows significant overall performance shortcomings for all the other transmitters that are located at greater distances. This is caused by the onset of sky wave propagation towards the evening, which superimposes the ground wave and causes strong distortion of the range estimation. This example was nevertheless selected in order to highlight the effect of the AGDF for the nearby Rozewie transmitter.

**Figure 7:**
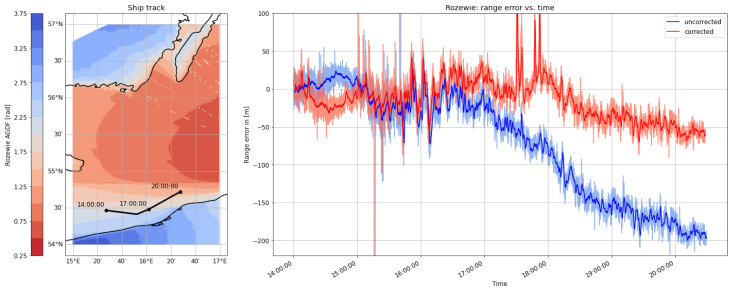
(**Left**) Track of the ship on **29 August 2020** on top of the AGDF map of the Rozewie MF R-Mode transmitter. (**Right**) Comparison of the range error for the Rozewie transmitter with AGDF (**red**) and without (**blue**). Light: raw samples; dark: average (60 s).

Taking a deeper look into the measurement of the Rozewie signal (Figure 7), the AGDF map for the area of interest shows a strong gradient in the phase delay due to the varying proportion of land with respect to the overall propagation path. The movement of the ship through this region causes a strong systematic bias in the range estimate, explaining the increased error and the multimodal distribution observed in the histogram. The movement of the vessel from a region of increased propagation delay though the gradient towards a region of smaller propagation delay explains the evolution of the range error in the negative direction. The compensation of the effect causes a smoothing of this systematic error trend towards zero. The overall noise level of the signal decreases with movement into the region of decreased ground wave distortion, as the received signal strength and signal-to-noise ratio naturally increase in this region as well.

The use of the predicted correction term improves the ranging accuracy significantly, and helps to improve the positional accuracy in the area.

**Figure 8:**
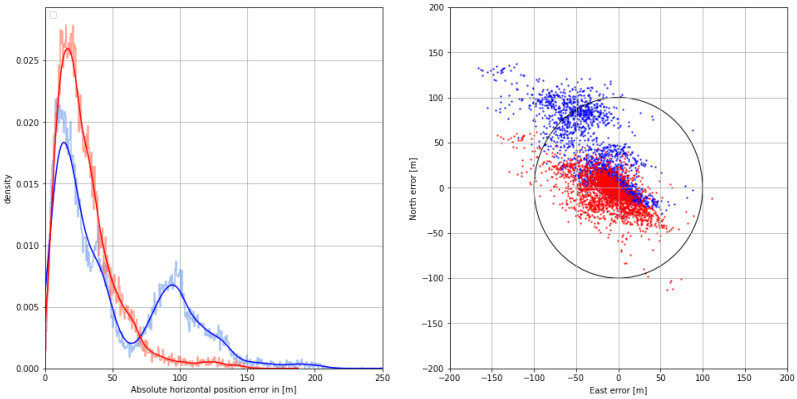
(**Left**) Histogram (pale color) and Gaussian kernel density estimate (firm color) of the corrected (**red**) and uncorrected (**blue**) horizontal position error on **29 August 2020**. (**Right**) Scatter plot of the corrected (**red**) and uncorrected (**blue**) 2D position error in the north and east directions. The circle represents the horizontal accuracy requirement of 100 m according to IALA R-129.

The distribution of the absolute horizontal position error with respect to the GNSS-based reference with dm accuracy is depicted in the graph section of Figure 8. The systematic bias caused by mixed land–sea ground wave propagation is compensated using the AGDF, increasing the overall position accuracy in that scenario. While the positioning performance is poor with respect to the 95% accuracy requirement of 100 m in the uncorrected case, the AGDF yields a result with sufficient accuracy and an improved standard deviation, as depicted in Table 4. The right side of Figure 8 shows the 2D horizontal position error in the east and north directions with respect to the GNSS-based reference with dm accuracy. Here, the uncorrected position estimate is biased to the northwest. The corrected position estimate is centered around an error of 10 m to the west. In this case, fewer measurements lie outside the 100 m error limit marked by the black circle.

#### 4.1.2. Second Dataset: Bay of Gdansk—30 August 2020

**Figure 9:**
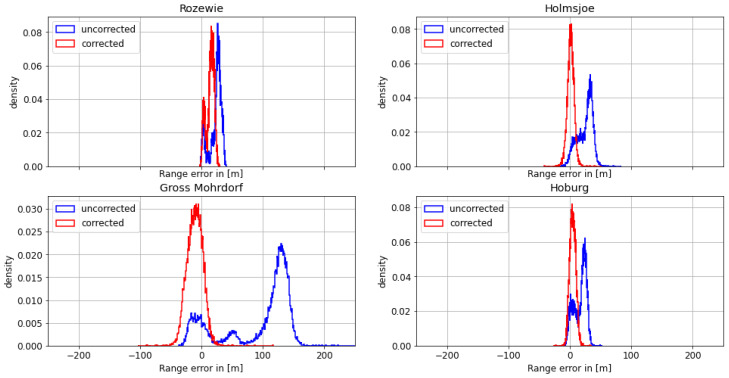
Histogram of the range error for the MF R-Mode transmitters in the testbed on **30 August 2020** with and without AGDF. **Blue**: no AGDF applied, ground wave phase delay not corrected. **Red**: AGDF applied, ground wave phase delay corrected.

**Figure 10:**
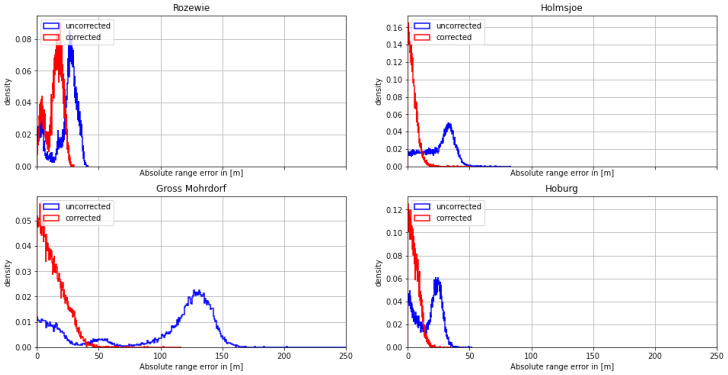
Histogram of the absolute range error for the MF R-Mode transmitters in the testbed on **30 August 2020** with and without AGDF. **Blue**: no AGDF applied, ground wave phase delay not corrected. **Red**: AGDF applied, ground wave phase delay corrected.

On the morning of August 30th, the ship continued its path along the Polish coast and turned south into the Bay of Gdansk. As depicted in Figure 9 and Figure 10, the range estimates of the R-Mode signals from Rozewie, Holmsjoe, and Hoburg were not significantly distorted by ground wave phase delays, as the propagation path consisted primarily of seawater and a small constant proportion of land in all three cases. The improvement is visible in the form of a shift of the density to the right, which can be attributed to the correct compensation of seawater propagation delay and the movement of the vessel away from all the transmitters. In the case of the Gross Mohrdorf signal, a multimodal distribution can be observed in the histogram of the range error, with three maxima around −10 m, 50 m, and 130 m. The systematic bias causes a large standard deviation and error in the 95th percentile (see Table 5).

**Figure 11:**
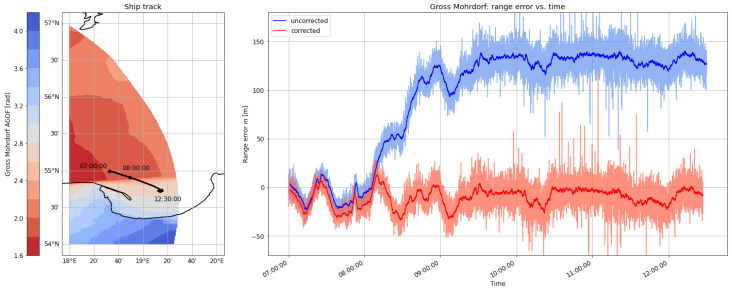
(**Left**) Track of the ship on **30 August 2020** on top of the AGDF map of the Gross Mohrdorf MF R-Mode transmitter. (**Right**) Comparison of the absolute of the range error for the Gross Mohrdorf transmitter with AGDF compensated (**red**) and without (**blue**). Light: raw samples; dark: average (60 s).

The systematic bias is caused by the increased phase delay due to the larger proportion of ground wave propagation over land when entering the bay. As in the previous example, the AGDF map in Figure 11 shows a strong gradient of AGDF along the ship track. The ship entered a region of increased distortion at around 8:00 GMT, causing a systematic bias towards 100 m range error. In contrast to the previous example, the error occurs in the positive direction due to opposite movement from a region of lower delay towards a region of increased distortion. This explains the increased noise level of the signal after entering the bay. The application of the phase-correction scheme compensates the systematic bias significantly (see also the histogram of the Gross Mohrdorf range error in Figure 9). The propagation path with an increased proportion of land after entering the bay causes a larger phase delay as well as a decrease in the signal strength, which directly affects the accuracy of phase estimation [36], explaining the larger standard deviation of the range error after 8:00 GMT.

**Figure 12:**
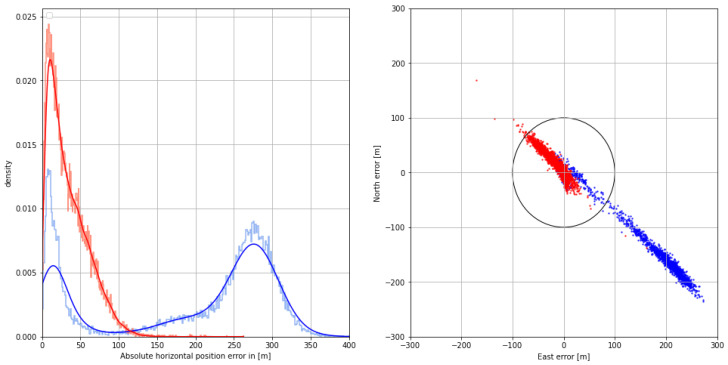
(**Left**) Histogram (pale color) and Gaussian kernel density estimate (firm color) of the corrected (**red**) and uncorrected (**blue**) horizontal position error on **30 August 2020**. (**Right**) Scatter plot of the corrected (**red**) and uncorrected (**blue**) 2D position error in north and east directions. The circle represents the horizontal accuracy requirement of 100 m according to IALA R-129.

The observed improvement in range level affects the position error as well. In addition to the influence of ground wave propagation, the geometry is relatively poor, with an HDOP of around 4. The histogram of the horizontal position error in Figure 12 shows that the systematic bias is compensated for with the AGDF error-correction scheme and that the overall position accuracy is better than 100 m in the 95th percentile (see Table 6). The scatter plot of the 2D horizontal position error in Figure 12 has an elongated, eccentric, and elliptical shape in both the corrected and uncorrected cases, which is oriented in the northwestern to southeastern direction due to the geometry of the transmitters, which are all located in either the western, northern, or northwestern direction. AGDF correction improves the performance significantly, with fewer measurements lying outside the 100 m error threshold.

#### 4.1.3. Third Dataset: Öland—31 August 2020

**Figure 13:**
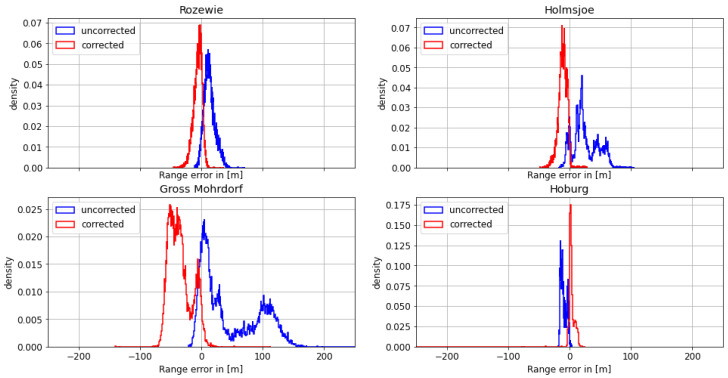
Histogram of the range error for the MF R-Mode transmitters in the testbed on **31 August 2020** with and without AGDF. **Blue**: no AGDF applied, ground wave phase delay not corrected. **Red**: AGDF applied, ground wave phase delay corrected.

**Figure 14:**
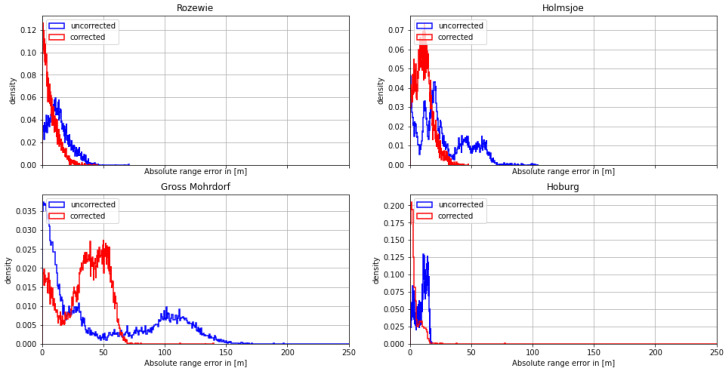
Histogram of the absolute range error for the MF R-Mode transmitters in the testbed on **31 August 2020** with and without AGDF. **Blue**: no AGDF applied, ground wave phase delay not corrected. **Red**: AGDF applied, ground wave phase delay corrected.

The measurement campaign concluded with a voyage back to the port of Stockholm. The ship travelled north along the coast of the Swedish island of Öland. While the signals transmitted from the MF stations in Rozewie and Hoburg travelled along a path with a relatively stable land–sea ratio, the proportion of the propagation path across land increased gradually in the case of the transmitted signals from Holmsjoe and Groß Mohrdorf during the maneuver. Figure 13 and Figure 14 displaythe histograms of the ranging error estimates and the absolute ranging error estimates for each MF R-Mode transmitter in view, depicting the uncorrected estimate in blue and the AGDF corrected estimate in red. In the cases of Rozewie and Hoburg, the mean error is shifted towards zero due to the correction of primarily seawater propagation delay. For Hoburg, the shift occurs to the right, as the vessel moved towards the transmitter, while it was moving away from the other transmitters, causing a shift to the left in these cases. The distribution of the error does not feature a significant bias, which indicates that the land–sea ratio of the propagation path did not change much. In the distribution of the ranging error of the signals from Groß Mohrdorf and Holmsjoe, a multimodality is clearly visible, as in the previously discussed maneuvers. In particular, the error distribution from Groß Mohrdorf has two maxima around 0 and 100 m. Table 7 shows that the AGDF correction improves the ranging accuracy significantly in all cases. Nevertheless, the error distribution of the Groß Mohrdorf signal is biased with AGDF corrections applied.

**Figure 15:**
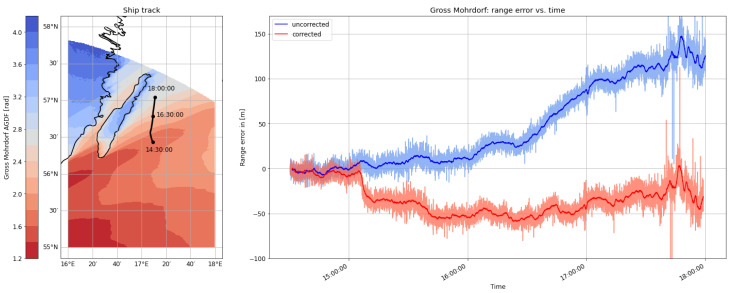
(**Left**) Track of the ship on **31 August 2020** on top of the AGDF map of the Gross Mohrdorf MF R-Mode transmitter. (**Right**) Comparison of the range error for the Gross Mohrdorf transmitter with AGDF (**red**) and without (**blue**). Light: raw samples; dark: average (60 s).

Figure 15 shows that the ship entered a region of increased ground wave phase delay due to propagation across the Swedish mainland and the island of Öland after 4 p.m. Though the AGDF correction helps to decrease the ranging error, there remains a systematic bias caused by overcompensation. In this particular case, the actual ground wave propagation delay is smaller than the predicted AGDF. One hypothesis that explains this effect is that the ground conductivity of the regions on land (e.g., Öland) is higher than the value obtained from the ITU World Atlas of Ground Conductivities. Alternatively, the salinity in this area of the Baltic Sea may have been much lower than assumed in the ITU-R 832-3, causing an overcompensation of the AGDF at the land–sea boundary. In general, the AGDF led to improved ranging performance.

**Figure 16:**
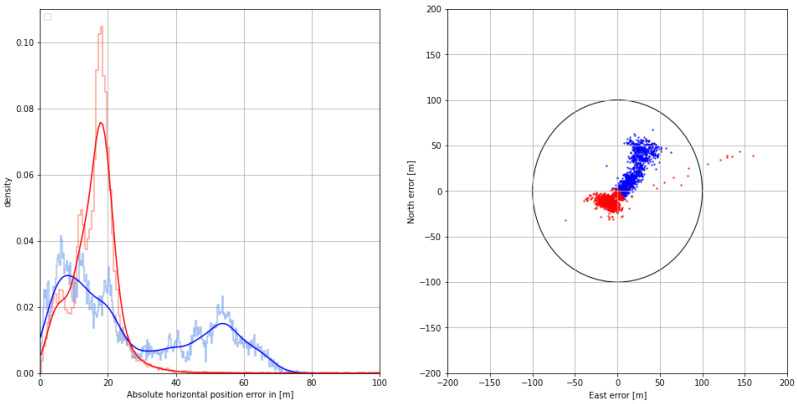
(**Left**) Histogram (pale color) and Gaussian kernel density estimate (firm color) of the corrected (**red**) and uncorrected (**blue**) horizontal position error on **31 August 2020**. (**Right**) Scatter plot of the corrected (**red**) and uncorrected (**blue**) 2D position error in north and east direction. The circle represents the horizontal accuracy requirement of 100 m according to IALA R-129.

With regard to the positioning performance, the AGDF corrected case exhibits a significant improvement over the uncorrected case. The error density shown in Figure 16 improves with the corrections, while the mean error, standard deviation, and 95th percentile error are all reduced (see Table 8). Nevertheless, the position is biased, with a mean error of around 17 m. While the position accuracy of both measurements is sufficient, AGDF correction improves the error density, as can be seen in the scatter plot of the 2D horizontal position error in Figure 16. Here, the uncorrected measurements have an elongated shape, while the corrected measurements are more densely distributed around the mean.

## 5. Discussion and Future Work

Medium-frequency R-Mode is a promising technology that can enable resilient PNT. The required positional accuracy [37] can be achieved if ground wave propagation delays are predicted and compensated.

The approach of using the Atmospheric and Ground Wave Delay Factor (AGDF) to correct ground wave phase delays has been introduced in this paper. The results obtained during a shipborne measurement campaign in the Baltic Sea R-Mode test bed clearly show improvement of the positional accuracy when AGDF correction is applied to the data. The datasets were obtained during maneuvers with increased impact of ground wave propagation due to variable land–sea propagation paths. In all of these cases, the accuracy of the R-Mode position was poor, sometimes even below the requirement of an error under 100 m 95% of the time. The AGDF decreases the ranging error and increases the positional accuracy significantly, allowing the system requirements to be fulfilled.

The AGDF approach is a method for predicting the complex attenuation of a wave at low and medium frequencies on a two-dimensional grid based on a database of ground conductivities. The approach is not limited to use within the MF R-Mode system, and can be applied to similar problems in the navigation domain. Though the prediction of ground wave propagation delays is always limited due to the lack of accurate real-time information on the propagation path, it yields results that are sufficiently accurate for the intended purpose. Even without extensive AGDF measurement surveys, the static prediction performs well in the Baltic Sea.

However, the results indicate that there are shortcomings in the proposed method. The data presented in this paper suggest that AGDF correction may yield values that are smaller or larger than the actual ground wave propagation delay in the area. A plausible explanation for this is discrepancy between the ground conductivity value obtained from the ITU World Atlas of Ground Conductivities and the actual value at the time of signal reception. The ground conductivity of an area depends on various factors, for example, the soil texture, volumetric water content, and temperature. At present, these parameters are not all covered by the static database provided by the ITU, although the authors have conducted initial investigations on that matter [38].

The AGDF prediction method employed in this paper lacks the ability to include the influence of higher-order propagation effects caused by terrain irregularities. Though this influence may be insignificant for certain smaller terrain features with respect to the wavelength of MF R-Mode signals, it has to be taken into account in the future.

The AGDF prediction method is not limited to the ground conductivity maps provided by the ITU. Future improvements will include the methods proposed in ITU-R P.527 and soil texture maps for selected areas. This will enable finer-grained prediction while taking moisture and temperature into account.

Another issue is the incorporation of the effect of sea ice into the prediction method. In combination with real-time sea ice coverage predictions, the AGDF could be refined for areas with significant ice coverage.

## Figures and Tables

**Table 1:** List of MF R-Mode transmitters in the Baltic Sea used during the campaign.

Transmitter	$f_{\mathrm{MSK}}$ [kHz]	$f_{\mathrm{CW}1}$ [kHz]	$f_{\mathrm{CW}2}$ [kHz]	Latitude	Longitude
Groß Mohrdorf (Germany)	308.0	307.775	308.225	54.374 ${}^{\circ }$N	12.934 ${}^{\circ }$E
Rozewie (Poland)	301.0	300.775	301.225	54.831 ${}^{\circ }$N	18.335 ${}^{\circ }$E
Hoburg (Sweden)	297.5	297.275	297.725	56.921 ${}^{\circ }$N	18.152 ${}^{\circ }$E
Holmsjoe (Sweden)	292.0	291.775	292.225	56.444 ${}^{\circ }$N	15.656 ${}^{\circ }$E

**Table 2:** Comparison of MF R-Mode and LORAN/eLoran with respect to selected system properties.

Property	MF R-Mode	Loran
Frequency	283–325 kHz	100 kHz
Access method	FDMA	TDMA
Signal type	CW	Pulsed and CW
Range estimation	TOA (signal phase/phase difference)	LORAN: TDOA, eLoran: TOA
Service area	250 km	up to 2000 km
Scope	Coastal areas, ports, inland waterways	Coastal areas, ports, open seas

**Table 3:** Absolute error $e_{range}$ of the range estimate with respect to GNSS-based reference track with dm accuracy reference track for each MF R-Mode transmitter.

Transmitter	Mean in [m]	σ in [m]	95% Error in [m]
Rozewie (not corrected)	77.0	67.5	188.7
Rozewie (corrected)	25.4	19.8	59.3
Gross Mohrdorf (not corrected)	15.8	16.3	50.9
Gross Mohrdorf (corrected)	22.2	20.6	65.6
Holmsjoe (not corrected)	26.4	30.7	84.8
Holmsjoe (corrected)	24.8	30.3	79.4
Hoburg (not corrected)	24.8	23.2	73.7
Hoburg (corrected)	14.5	14.0	44.2

**Table 4:** Horizontal error $e_{pos}$ of the position estimate with respect to GNSS-based reference track with dm accuracy.

Phase Delay	Mean in [m]	σ in [m]	95% Error in [m]
(not corrected)	50.4	43.1	128.9
(corrected)	30.3	22.7	71.6

**Table 5:** Absolute error $e_{range}$ of the range estimate with respect to GNSS-based reference track with dm accuracy for each MF R-Mode transmitter.

Transmitter	Mean in [m]	σ in [m]	95% Error in [m]
Rozewie (not corrected)	23.4	10.0	35.2
Rozewie (corrected)	14.5	6.5	23.3
Gross Mohrdorf (not corrected)	96.4	50.2	145.8
Gross Mohrdorf (corrected)	13.2	9.9	31.4
Holmsjoe (not corrected)	25.2	11.9	40.7
Holmsjoe (corrected)	4.4	3.6	11.1
Hoburg (not corrected)	17.1	9.6	29.8
Hoburg (corrected)	5.6	3.9	12.7

**Table 6:** Horizontal error $e_{pos}$ of the position estimate with respect to GNSS-based reference track with dm accuracy.

Phase Delay	Mean in [m]	σ in [m]	95% Error in [m]
(not corrected)	190.8	115.8	315.5
(corrected)	34.3	25.9	84.0

**Table 7:** Absolute error $e_{range}$ of the range estimate with respect to GNSS-based reference track with dm accuracy for each MF R-Mode transmitter.

Transmitter	Mean in [m]	σ in [m]	95% Error in [m]
Rozewie (not corrected)	13.6	8.6	30.3
Rozewie (corrected)	6.8	5.8	18.3
Gross Mohrdorf (not corrected)	47.4	46.0	125.7
Gross Mohrdorf (corrected)	34.9	17.7	58.8
Holmsjoe (not corrected)	26.2	19.0	61.2
Holmsjoe (corrected)	11.1	6.7	23.1
Hoburg (not corrected)	9.6	4.7	15.6
Hoburg (corrected)	6.1	19.4	13.4

**Table 8:** Horizontalerror $e_{pos}$ of the position estimate with respect to GNSS-based reference track with dm accuracy.

Phase Delay	Mean in [m]	σ in [m]	95% Error in [m]
(not corrected)	26.6	20.2	61.9
(corrected)	16.9	14.1	26.0

**Figure A1:**
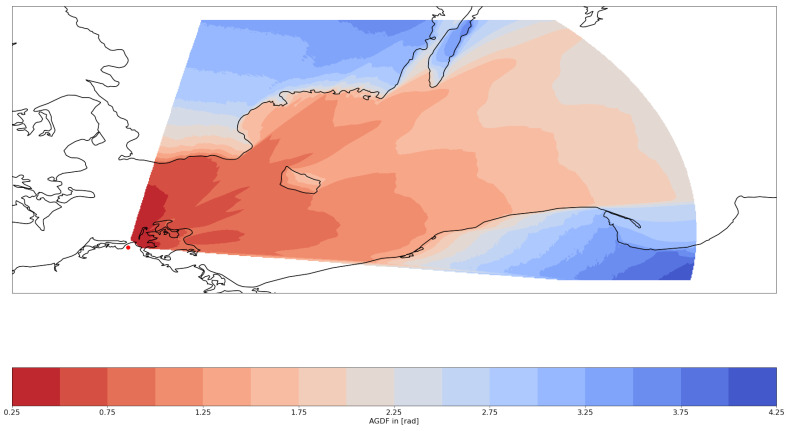
Predicted AGDF map for the Groß Mohrdorf MF R-Mode transmitter. The transmitter location is indicated by the red dot.

**Figure A2:**
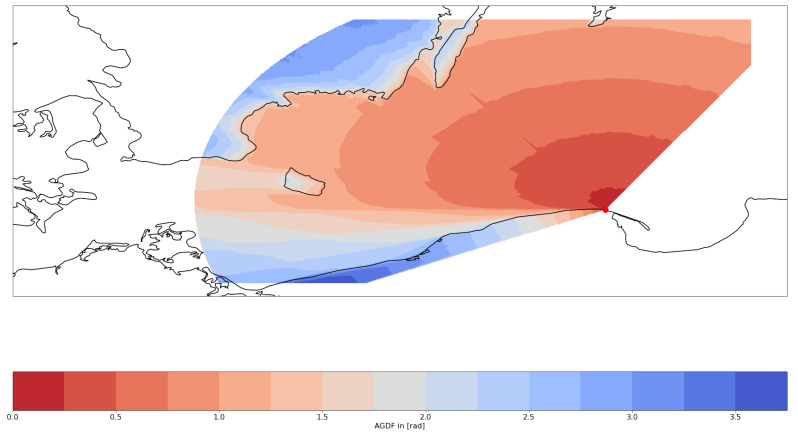
Predicted AGDF map for the Rozewie MF R-Mode transmitter. The transmitter location is indicated by the red dot.

**Figure A3:**
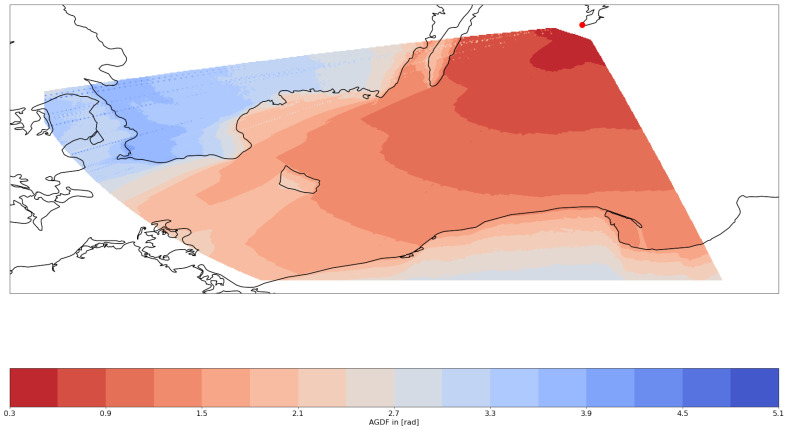
Predicted AGDF map for the Hoburg MF R-Mode transmitter. The transmitter location is indicated by the red dot.

**Figure A4:**
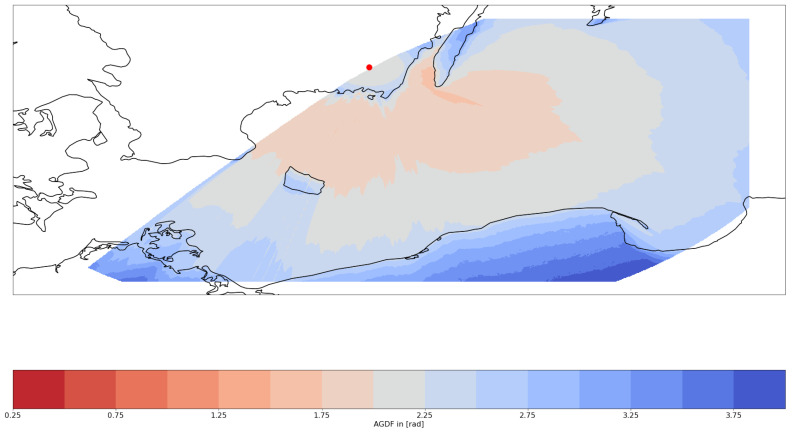
Predicted AGDF map for the Holmsjoe MF R-Mode transmitter. The transmitter location is indicated by the red dot.

## Data Availability

Data are contained within the article.

## Abbreviations

The following abbreviations are used in this manuscript:

R-Mode	Ranging-Mode
AGDF	Atmospheric and Ground wave Delay Factor
MF	Medium Frequency
CW	Continuous Wave
GNSS	Global Navigation Satellite System(s)
PNT	Position, Navigation, and Timing
VDES	Very-High-Frequency Data Exchange System
FDTD	Finite Difference Time Domain
FEM	Finite Element Method
MOM	Method of Moments
EM	Electromagnetic
ITU	International Telecommunication Union
HDOP	Horizontal Delusion of Precision
ASF	Additional Secondary phase Factor
PF	Primary phase Factor
SF	Secondary phase Factor
TDMA	Time-Division Multiple Access
FDMA	Frequency-Division Multiple Access
TDOA	Time Difference of Arrival
TOA	Time of Arrival
PPP	Precise Point Positioning
